# Supplementary material for: Faster Rates of Molecular Sequence Evolution in Reproduction-Related Genes and in Species with Hypodermic Sperm Morphologies
Source: Mol Biol Evol. 2021 Sep 17;38(12):5685–703. doi: 10.1093/molbev/msab276 (PMC8662610; doi:10.1093/molbev/msab276)

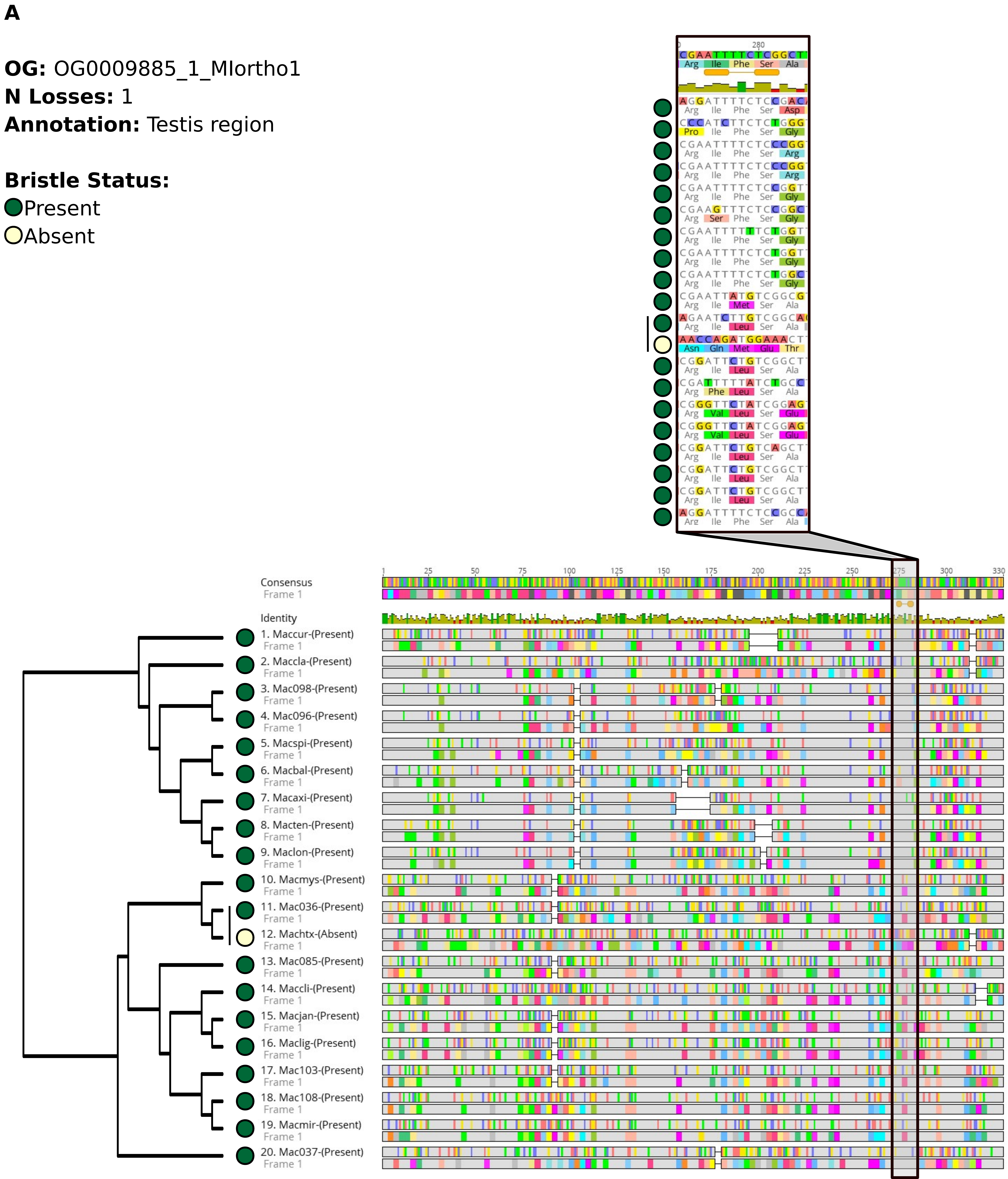
A
OG: OG0009885_1_MIortho1
N Losses: 1
Annotation: Testis region
Bristle Status:
Present
Absent
Consensus
Frame 1
Identity
1. Maccur-(Present)
2. Maccla-(Present)
3. Mac098-(Present)
4. Mac096-(Present)
5. Macspi-(Present)
6. Macbal-(Present)
7. Macaxi-(Present)
8. Macten-(Present)
9. Maclon-(Present)
10. Macmys-(Present)
11. Mac036-(Present)
12. Machtx-(Absent)
13. Mac085-(Present)
14. Maccli-(Present)
15. Macjan-(Present)
16. Maclig-(Present)
17. Mac103-(Present)
18. Mac108-(Present)
19. Macmir-(Present)
20. Mac037-(Present)
Frame 1
1
25
50
75
100
125
150
175
200
225
250
275
300
330
280
CGAATTTTCTCGGCT
Arg Ile Phe Ser Ala

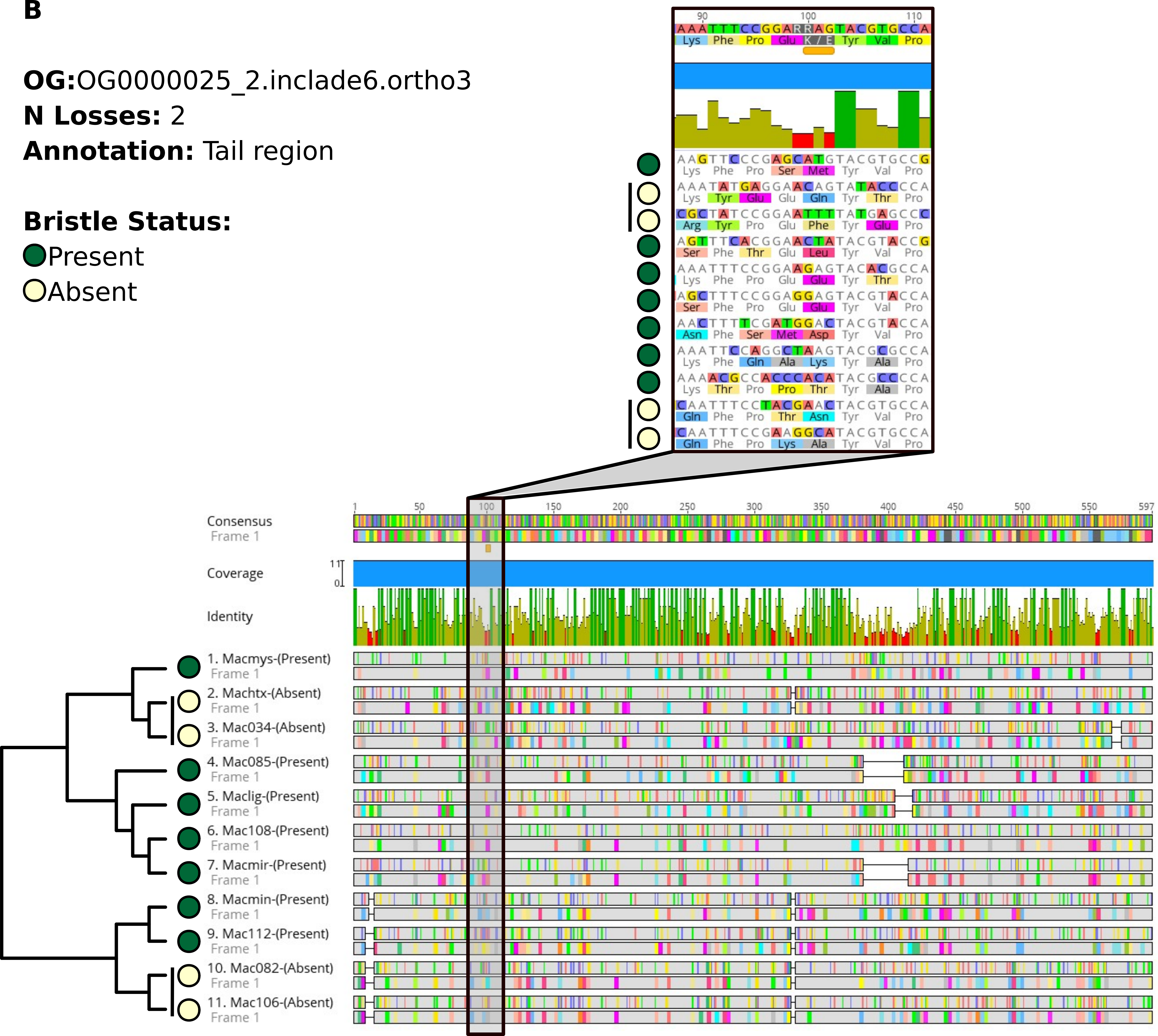

B
OG:OG0000025_2.inclade6.ortho3
N Losses: 2
Annotation: Tail region
Bristle Status:
Present
Absent
Consensus
Frame 1
Coverage
Identity
1. Macmys-(Present)
2. Machtx-(Absent)
3. Mac034-(Absent)
4. Mac085-(Present)
5. Maclig-(Present)
6. Mac108-(Present)
7. Macmir-(Present)
8. Macmin-(Present)
9. Mac112-(Present)
10. Mac082-(Absent)
11. Mac106-(Absent)

C

OG:OG0000285_1.inclade1.ortho7
N Reductions: 4
Annotation: Testis region

Bristle Status:
Present
Reduced

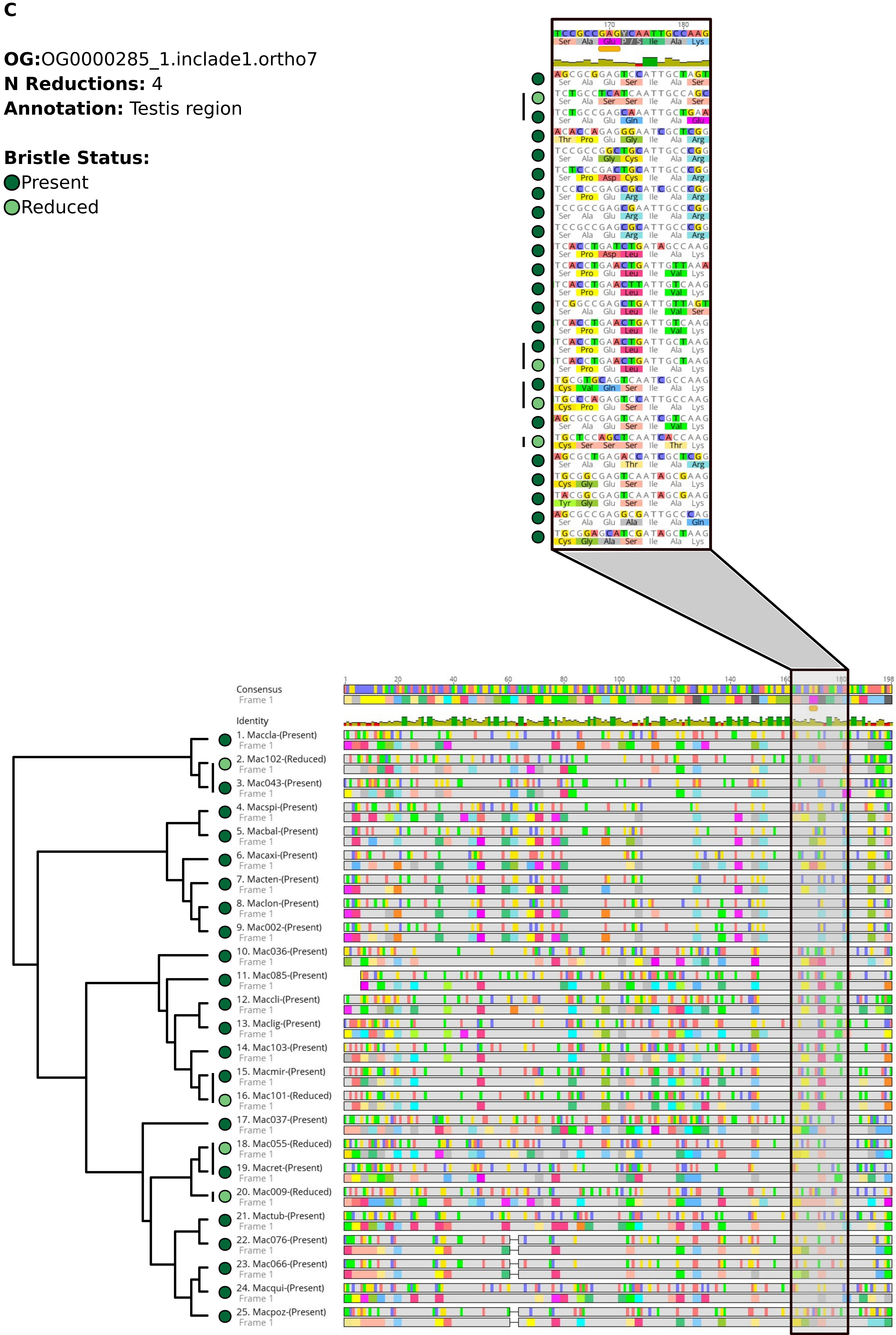

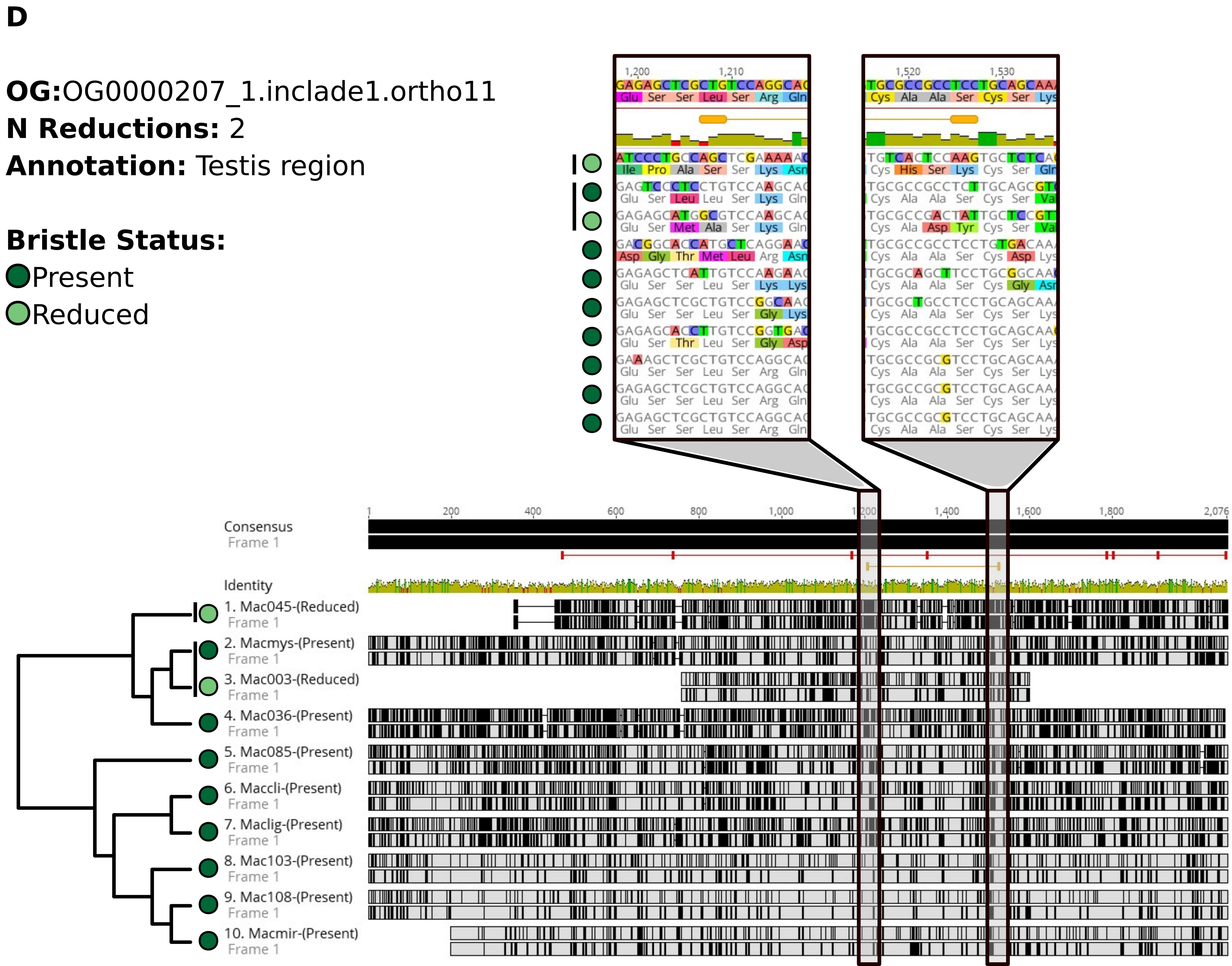
D
OG:OG0000207_1.inclade1.ortho11
N Reductions: 2
Annotation: Testis region
Bristle Status:
Present
Reduced
Consensus
Frame 1
Identity
1. Mac045-(Reduced)
2. Macmys-(Present)
3. Mac003-(Reduced)
4. Mac036-(Present)
5. Mac085-(Present)
6. Maccli-(Present)
7. Maclig-(Present)
8. Mac103-(Present)
9. Mac108-(Present)
10. Macmir-(Present)
1 200 400 600 800 1,000 1,200 1,400 1,600 1,800 2,076
1,200 1,210
1,520 1,530

E

OG:OG0000247_1.inclade3.ortho5

N Losses: 3

Annotation: Testis region

Bristle Status:

Present

Absent

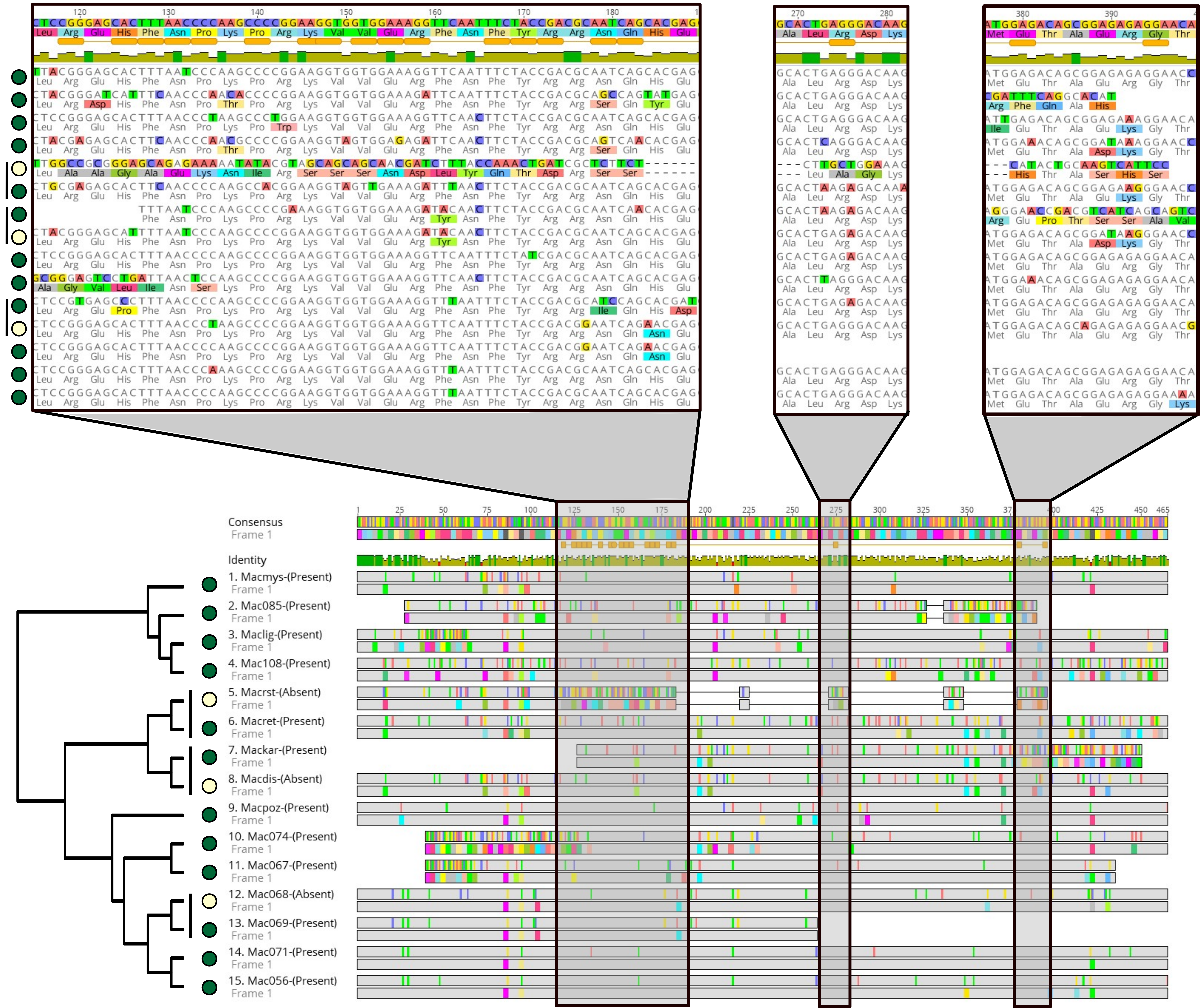

F

OG:OG0001675_2_MIortho3
N Losses: 1
Annotation: Ovary region

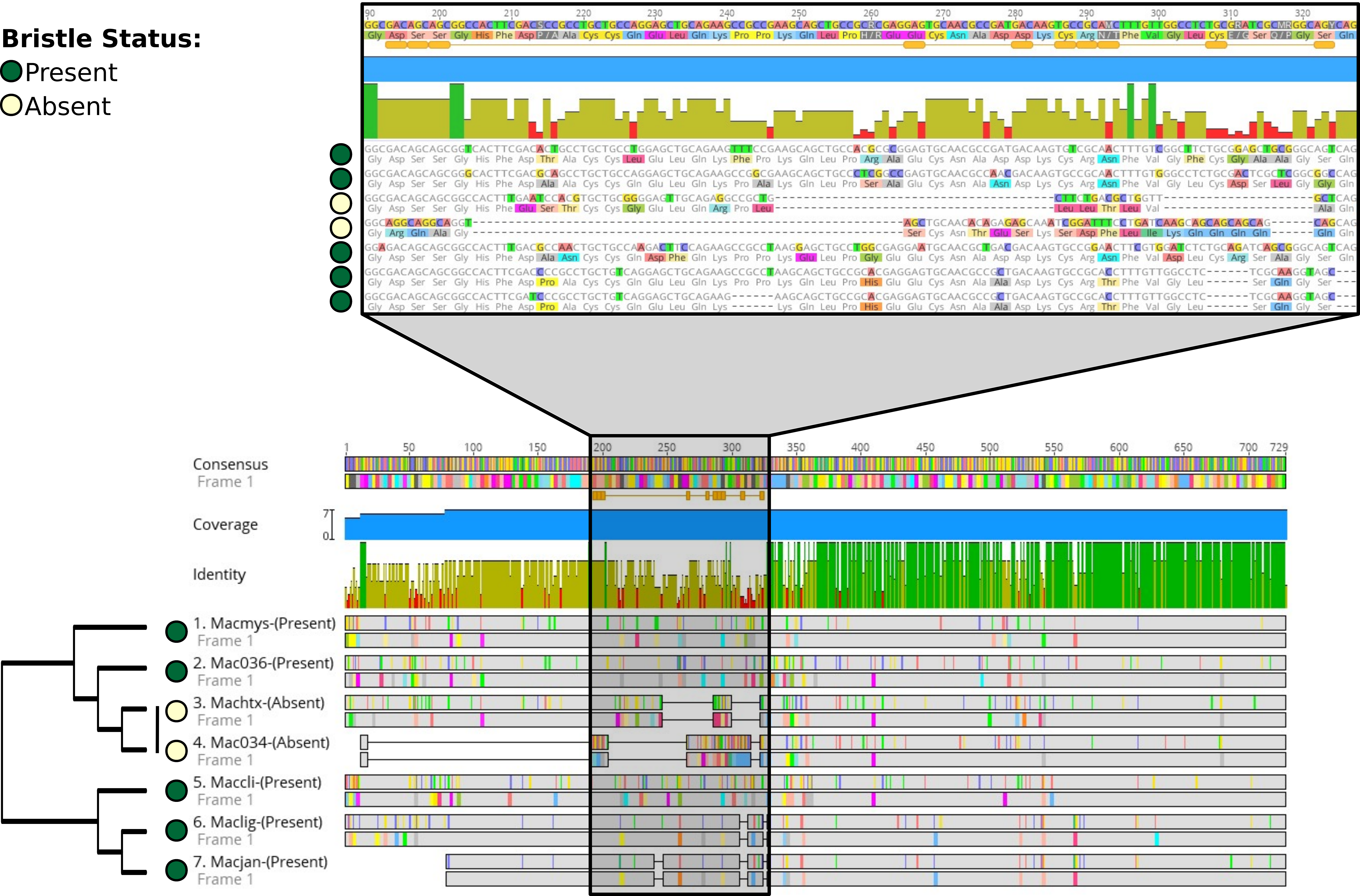

G

OG:OG0000113_4.inclade1.ortho5

N Reductions: 1

Annotation: Ubiquitously expre

Bristle Status:

Present

Reduced

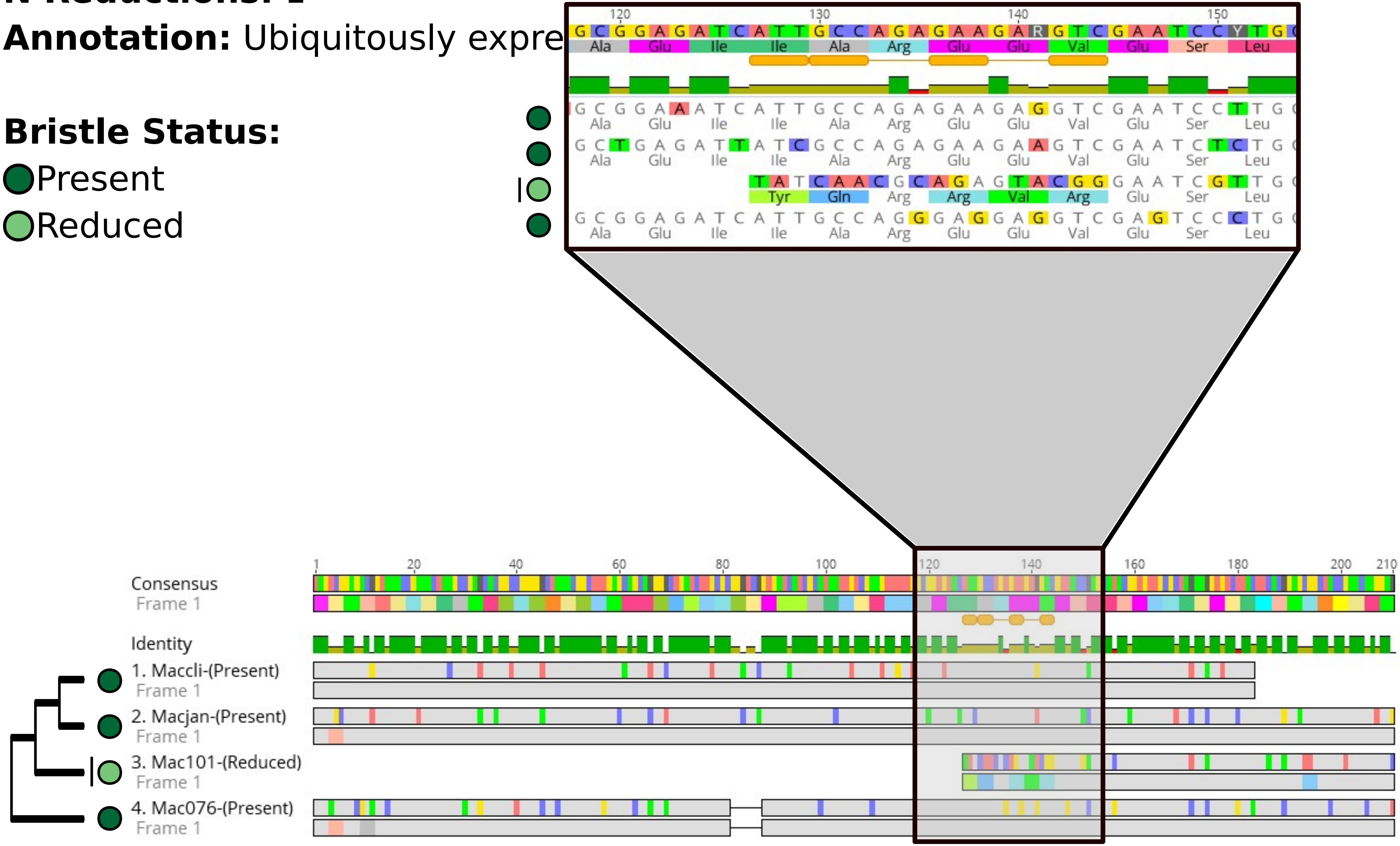

H

OG:OG0011654_1_MIortho1
N Losses: 5
Annotation: Ovary region

Bristle Status:
Present
Absent

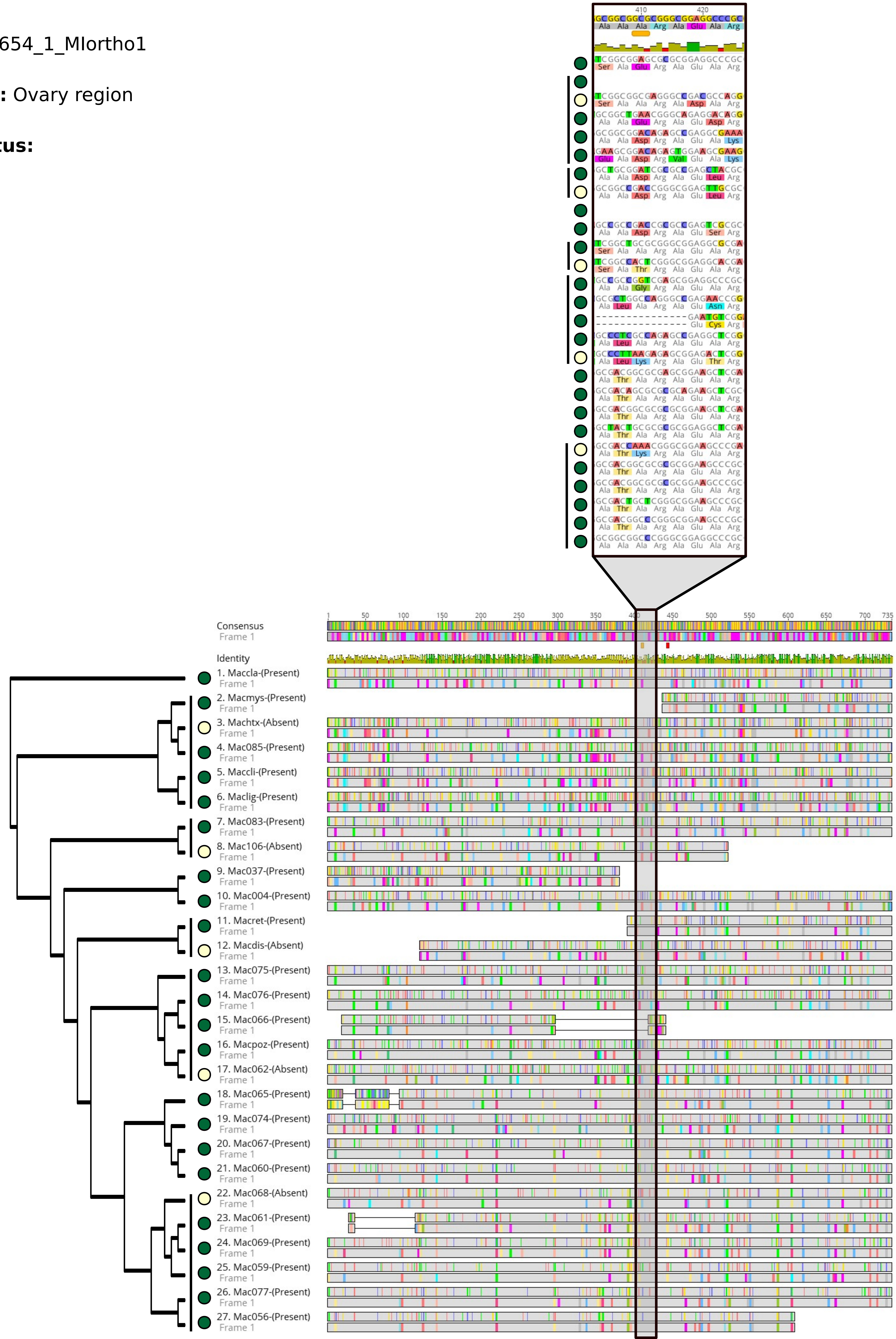

I

OG:OG0000222_1.inclade1.ortho11

N Losses: 2

Annotation: Ovary region

Bristle Status:

Present

Absent

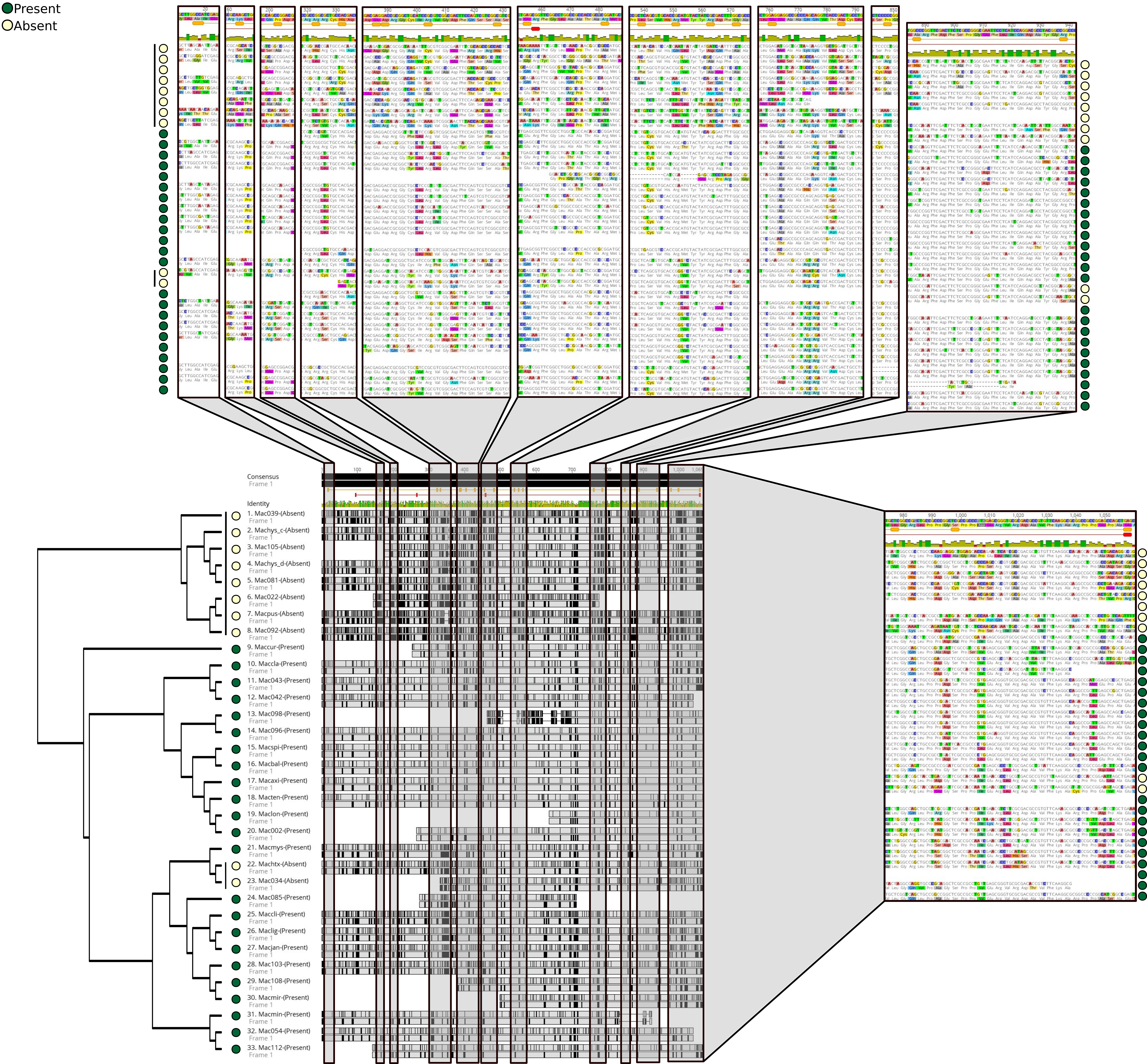

J

OG:OG0003507_1.inclade1.ortho3

N Reductions: 2

Annotation: Tail region

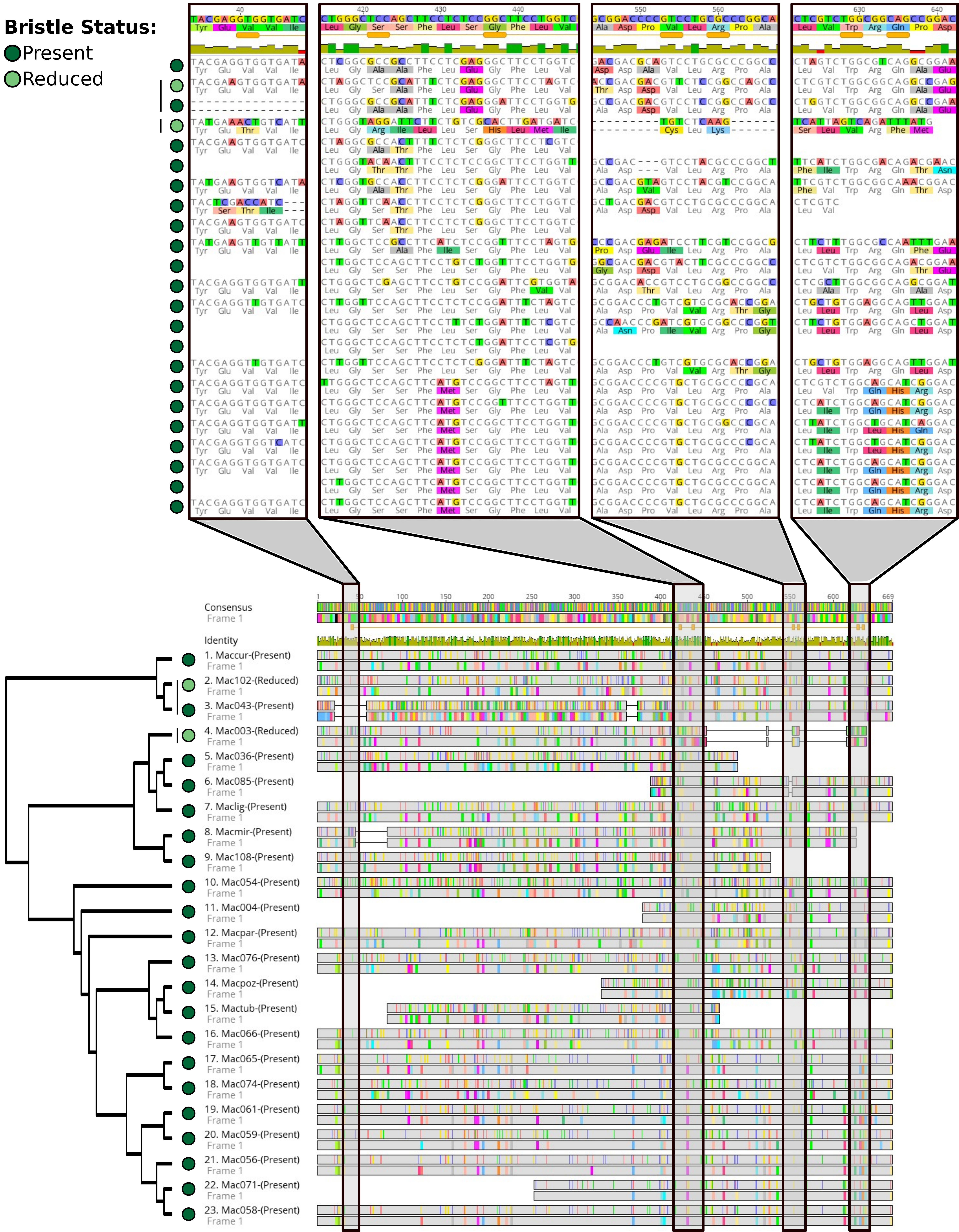

Supplement: msab276_Supplementary_Data [file msab276_supplementary_data.zip › FigS8.pdf]
