# Supplementary figures and images for: Faster Rates of Molecular Sequence Evolution in Reproduction-Related Genes and in Species with Hypodermic Sperm Morphologies
Source: Mol Biol Evol. 2021 Sep 17;38(12):5685–703. doi: 10.1093/molbev/msab276 (PMC8662610; doi:10.1093/molbev/msab276)

Hypothesis Test (Alt.

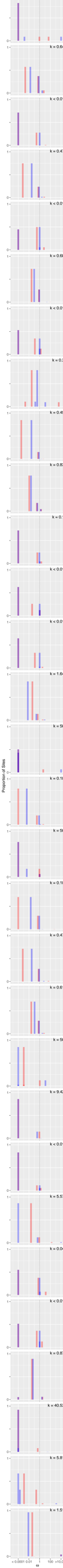

### Partitioned Description

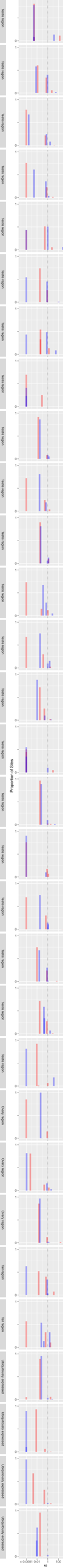

Supplement: msab276_Supplementary_Data [file msab276_supplementary_data.zip › FigS10.pdf]

Hypothesis Test (Alt.

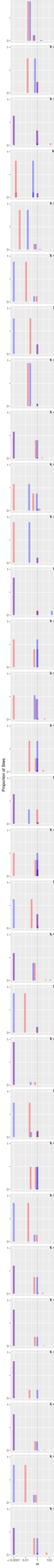

Model) Partition

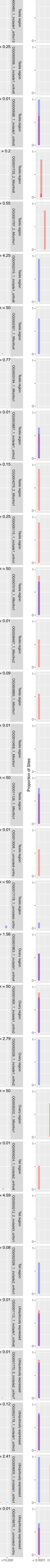

Supplement: msab276_Supplementary_Data [file msab276_supplementary_data.zip › FigS11.pdf]
